# Implicit race attitudes modulate visual information extraction for trustworthiness judgments

**DOI:** 10.1371/journal.pone.0239305

**Published:** 2020-09-24

**Authors:** Isabelle Charbonneau, Karolann Robinson, Caroline Blais, Daniel Fiset

**Affiliations:** Groupe de Neurosciences Sociales, Département de Psychoéducation et de Psychologie, Université du Québec en Outaouais, Gatineau, QC, Canada; Institut VEDECOM, FRANCE

## Abstract

Black people are still considered to be one of the most stigmatized groups and have to face multiple prejudices that undermine their well-being. Assumptions and beliefs about other racial groups are quite pervasive and have been shown to impact basic social tasks such as face processing. For example, individuals with high racial prejudice conceptualize other-race faces as less trustworthy and more criminal. However, it is unknown if implicit racial bias could modulate even low-level perceptual mechanisms such as spatial frequency (SF) extraction when judging the level of trustworthiness of other-race faces. The present study showed that although similar facial features are used to judge the trustworthiness of White and Black faces, own-race faces are processed in lower SF (i.e. coarse information such as the contour of the face and blurred shapes as opposed to high SF representing fine-grained information such as eyelashes or fine wrinkles). This pattern was modulated by implicit race biases: higher implicit biases are associated with a significantly higher reliance on low SF with White than with Black faces.

## 1. Introduction

From a simple look at a face, it is possible to obtain a wealth of information such as race, gender, age or emotional state [1,2]. One of the crucial attributes that humans quickly extract from faces is trustworthiness [3–6]; in fact, this judgment can be made with a face viewed for as little as 34 milliseconds [7]. Strikingly, even with faces viewed for a very short duration, individuals show a high interindividual agreement in their face trustworthiness judgments [8,9].

Studies show that these judgments are biased by the appearance of faces. A neutral face showing slight signs of happiness, a facial expression strongly associated with changes in mouth shape [10], will be judged as more trustworthy [8,11]. In contrast, a neutral face in which facial features slightly overlap with those associated with anger, for instance V-shaped eyebrows and a high contrasted fold between eyebrows [12], will be deemed relatively untrustworthy [8,11]. Moreover, the spatial resolution in which such facial features are visually processed has an important role to play [13]. In psychophysics, spatial resolution is represented by spatial frequencies (SF), where high SF represent the fine-grained information in a stimulus, such as eyelashes or fine wrinkles, and low SF convey coarse information, such as the contour of the face and blurred shapes (see [14]). Processing the eye region in high SF and the mouth area in a broad range of SF positively influences faces’ trustworthiness rating [13]. Furthermore, processing the whole face in low SF also increased trustworthiness ratings [13], possibly because low SF make the skin appear smoother [13] and the face shape appears rounder and more baby-like [15]. Interestingly, it was shown that keeping the shape of features constant but manipulating their SF content can predictably rig the way a face will be evaluated, by either increasing the trustworthiness percept or decreasing it. These results therefore suggest that the facial information we pay attention to can modulate our interpretation of faces and, in turn, influence our perception of trustworthiness.

Unfortunately for individuals who possess facial characteristics perceived as untrustworthy, these judgments have a strong impact on how other people behave with them [16,17]. For example, people with untrustworthy-looking faces attract less financial investment in both ecological and laboratory settings [18,19], are more prone to be declared as criminally guilty [20] and receive harsher criminal sentences [21]. Those outcomes are troubling as they are not justified by any proven link between perceived and real trustworthiness [22] (see [23] for a discussion of this topic).

Strikingly similar observations were also made for visible minorities such as Black people in Westernized nations. As an example, members of this racial group are more frequently wrongfully convicted of a crime as a result of eyewitness identification both in the US [24] and in other countries [25], and their sentences tend to be more severe [26]. Black people are twice as likely to be unemployed as Whites [27], and evidence suggests that they face higher rejection rates and less favorable terms in securing mortgages than do Whites with a similar credit history [28].

Relatedly, research suggests a relationship between trustworthiness judgments and implicit racial biases; trustworthiness estimations and propensity to trust during an economic game are both modulated by the race of the face and this effect is stronger for participants with a larger pro-White implicit bias [29]. In fact, assumptions and beliefs about another racial group are quite pervasive and have been shown to impact even basic tasks such as racial categorization. For instance, Dotsch et al., [30] showed that prejudices entail bias in the way people conceptualize the facial appearance of people from another racial group. Individuals with a high level of racial prejudice conceptualize other-race faces as less trustworthy and more criminal. In line with this, other studies have shown that higher implicit prejudices in White observers are associated with a greater readiness to perceive anger in Black faces [31]. Since, as explained above, features connoting anger are associated with low trustworthiness ratings [8], these findings may reflect a tendency, in prejudiced individuals, to rely on different facial features during the processing of Black and White faces. For instance, it is possible that prejudiced individuals pay more attention to features connoting anger in Black faces while they instead pay more attention to features connoting happiness in White faces. This would increase the likeliness of perceiving anger in Black faces, which would in turn affect trustworthiness judgments. If prejudiced individuals pay attention to different facial features in Black and White faces during trustworthiness judgments, it is also possible that they pay attention to different SF. As mentioned above, attending to low SF increases the percept of trustworthiness, plausibly by making the skin texture very smooth and by increasing the baby-likeness of the face shape.

Therefore, in the present study, we will support the hypothesis that implicit racial biases may affect the visual information on which individuals rely to decide whether a Black versus a White face looks trustworthy. More specifically, we hypothesize that racially prejudiced individuals will rely more on features associated with anger and less on features associated with happiness, and make lesser use of low SF, for Black than for White faces.

## 2. Methods and results

**Fig 1:**
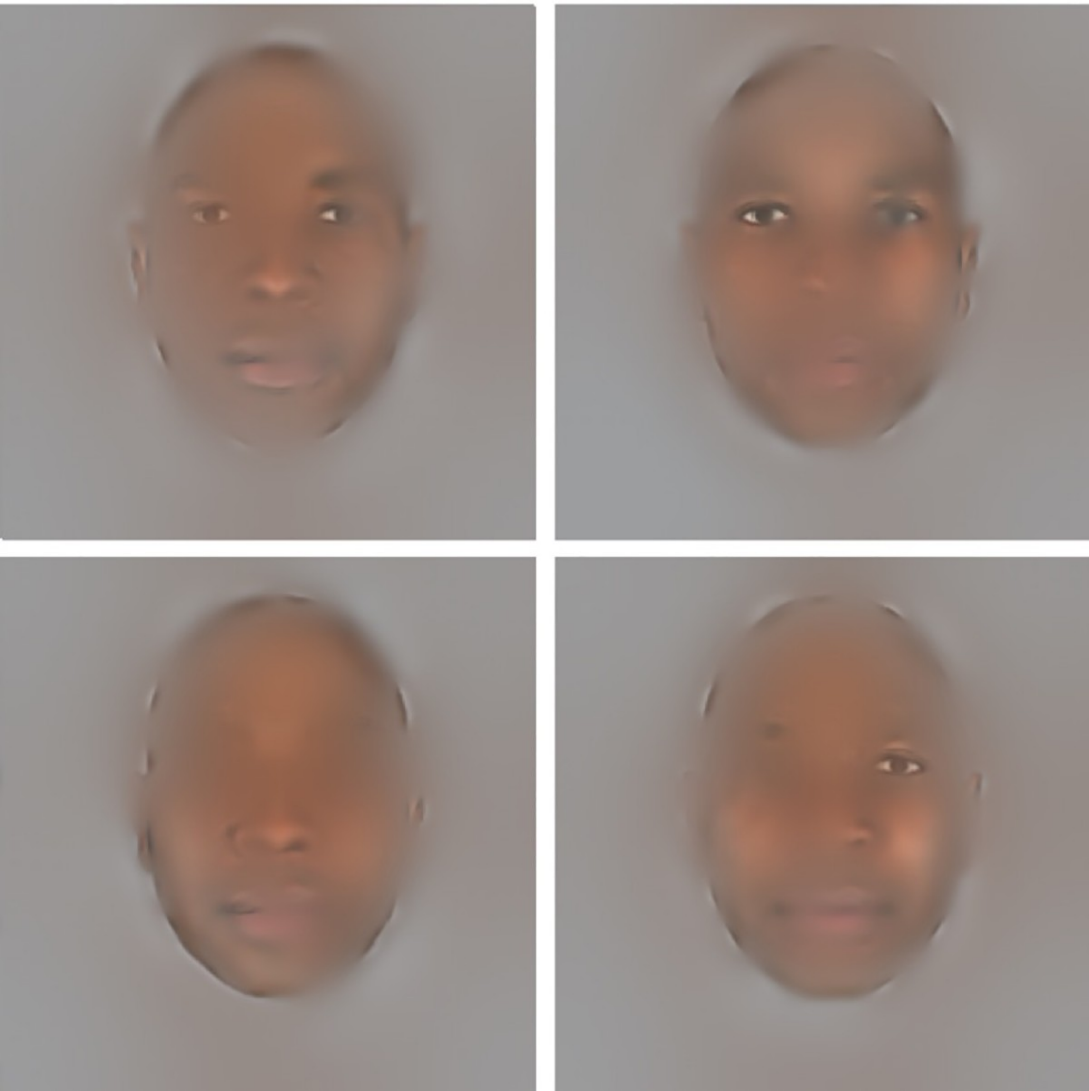
Four examples of stimuli created using the Bubbles method. Using this method, facial areas and the SF bands in which they are revealed vary from one trial to the other. Across trials, it is possible to infer which features in which SF increased the probability of answering correctly. Note that the face stimuli used in this and all following figures are not part of the database used in the reported study, for copyright reasons; they were instead taken from a database of artificially generated faces under a CC BY license, with permission from [34,35]; http://tlab.princeton.edu/databases/). Facial features in our examples have been precisely aligned in order to best represent the average position of features from our stimuli for both Black and White faces.

In the present study, the Bubbles method [32] was used to pinpoint the perceptual information used by an observer to successfully decide which of two faces is perceived as the most trustworthy. The general idea of the Bubbles method is to randomly manipulate the visual information available in a face stimulus to infer which information is correlated with success in the task at hand. For example, during a face identification task, if the eyes are important (i.e. are diagnostic; see [33]), their presence in the stimulus (see Fig 1 for examples) will often lead to a correct answer. On the other hand, if the region of the nose is not diagnostic for the task, its presence in the Bubblized stimulus will not change the probability of a correct answer. Thus, by using Black and White Bubblized faces, it is possible to compare the visual information on which participants rely to make a judgment of trustworthiness with each racial group of faces. Fig 1 provides an example of the type of stimulus produced using that method. In the present experiment, and as displayed in Fig 1, visual information in terms of facial parts and SF will be manipulated. Thus, on each trial, different parts of the face in different SF will be selected and presented to the participant, while the rest of the visual information will remain hidden. For instance, in the examples provided in Fig 1, the eyes are available in the stimuli presented in the top row, but they are not available in the stimulus displayed in the bottom left panel, and only the right eye is available in the stimulus displayed in the bottom right panel. Moreover, the resolution of the visual information available in the eye area differs across these four stimulus examples: while the stimulus displayed on the bottom right panel has a high resolution in the eye area, the right eye of the stimuli displayed in the top row have a lower resolution.

One of the main advantages of the Bubbles method is that it highly reduces the need of making *a priori* decisions regarding which information may or may not be important for a given task. For instance, to measure which face parts are used by participants to discriminate trustworthiness level, Bubbles randomly sample among pixels of a face on each trial; therefore, one does not need to make arbitrary decisions about what are the important features in a face, what size and resolution they should have, and so on.

The core experiment of the present study, namely the application of Bubble filters to faces, was preceded by two phases. First, in order to measure what visual information allows to discriminate faces based on their trustworthiness, we needed to create pairs of faces that differed in their level of perceived trustworthiness. The creation of such pairs was the aim of Phase 1. To achieve this, ratings were collected to measure how each face included in the final face set was perceived on average by a group of individuals. Based on these ratings, three categories were created: “high”, “neutral”, and “low” trustworthiness. Those categories were then used to create pairs composed of one face from the “high trustworthiness” category and another from the “low trustworthiness” category.

The second Phase aimed at verifying if it was possible to achieve a reasonably high accuracy at evaluating the trustworthiness of the faces included in the pairs created in Phase 1. This verification was made to ensure that participants were able to correctly discriminate between a trustworthy and untrustworthy face prior to Phase 3 since the Bubbles method decreases the amount of visual information available to do the same task.

Finally, in the third Phase, which represented the core of the present study, the pairs of faces used in Phase 2 were presented to participants, but this time while being sampled using the Bubbles method. At the end of the third Phase, an Implicit Association Test (IAT; [36]) was administered to all participants to measure their level of implicit racial bias towards Black versus White individuals. Note that in the present study, we report all measures, manipulations and exclusions. Informed consent was obtained from all participants; all procedures were carried out in accordance with Université du Québec en Outaouais’s Ethics Guidelines and were approved by the Université du Québec en Outaouais’s Research Ethics Committee. Note that task instructions, verbal content, and the IAT were presented to participants in their native language (i.e. french).

### 2.1 Stimuli and materials

The stimuli consisted of 329 faces, including 184 White faces and 145 Black faces [37]. To avoid any form of bias during the selection of stimuli, all identities of the image bank were kept, with the exception of 4 duplicates. All pictures depicted a male face viewed from the front, with a neutral expression and open eyes. The pictures were spatially aligned with the positions of the main internal facial features (eyes, mouth, and nose) using translation, rotation, and scaling manipulation. Importantly, these manipulations do not modify relative distances between features of the face (e.g. distances between the two eyes and between the eyes and the mouth). Face width subtended 7 degrees of visual angle on average. This face size was chosen in keeping with previous studies showing that expert face processes take place for faces larger than 6 degrees of visual angle [38].

All stimuli were displayed in color on a 22-inch Samsung LED monitor with a refresh rate of 120 Hz. The experiment ran on an Apple MacPro QuadCore computer. The experimental program was written in Matlab, using the Psychophysics Toolbox [39,40].

### 2.2 Phase 1

#### 2.2.1 Participants

A group of 40 participants (mean age of 24.1 years old, 29 women and 11 men) was recruited. All participants were European-Canadian and had corrected-to-normal visual acuity.

#### 2.2.2 Procedure

The experiment consisted of two consecutive blocks comprising either White or Black faces. The order in which the blocks were administered was counterbalanced across participants, such that half of them started with Black faces, and the other half started with White faces. On each trial, the participants’ task was to judge a face’s level of trustworthiness on a scale that ranged from 1 (*very untrustworthy)* to 9 (*very trustworthy*). Participants were told to rely on their gut feeling and that there was no right or wrong answer. The faces were presented in random order.

#### 2.2.3 Analysis and results

Anonymized datasets for all experiments are available at the following URL: https://osf.io/x6n25/. For each participant and each racial group of face stimuli, we first z-scored the ratings to take into account individual differences in the use of the scale. We then calculated the average rating across participants for each face in order to obtain a reliable measure of their level of trustworthiness. We used the average rating since this measure predicts the brain's response to trust (i.e. increased amygdala response as perceived trustworthiness decreases [41]). Average correlation between individual judgments and the mean for the remaining individuals across the 329 identities was *r* = 0.45. This result is slightly lower but consistent with the same analysis performed by Engell et al., [41] (*r* = .52). The average correlation between individual judgments and the mean for the remaining individuals was *r* = 0.43 for White and *r* = 0.47 for Black faces. Based on these judgments, we then divided the faces into three categories for each racial group: the 50 faces with the highest trustworthiness level, the 50 faces with the lowest trustworthiness level, and the other faces considered neutral or difficult to appraise with respect to this trait. The two extreme categories (highest and lowest trustworthiness level) were then used in the second Phase to assess whether naive participants were able to accurately distinguish between two faces, one taken from the “high trustworthiness” category and one taken from the “low trustworthiness” category.

Additional analyses were conducted in order to investigate potential differences in the way White and Black faces’ trustworthiness was judged. A two-tailed paired t-test was conducted on the raw trustworthiness ratings for White (*M* = 4.70, *SD* = 0.82) and Black (*M* = 4.56, *SD* = 0.91) faces; there was no significant difference (*t*(327) = 1.45, *p* = 0.15, *Cohen’s d* = 0.15, 95% CI [-0.33 0.05].

Finally, a two-way ANOVA on the factors of race (White vs. Black) and trustworthiness group (“low” vs. “high”) was conducted to verify if there were differences in the average rating of “low trustworthiness” vs. “high trustworthiness” faces as a function of race. The effect of trustworthiness was significant (*F*(1, 196) = 1213.6, *p*<0.001, η^2^ = 0.859). The effect of race was not significant (*F*(1, 196) = 3.6, *p* = 0.06, η^2^ = 0.003): while it was nearly significant, the effect size was very small. Importantly, the interaction between both factors was not significant (*F*(1, 196) = 0.38, *p*<0.54, η^2^<0.001).

### 2.3 Phase 2

The aim of Phase 2 was to verify if the trustworthiness categories created in Phase 1 allowed participants to achieve a good performance at discriminating faces based on their trustworthiness level.

#### 2.3.1 Participants

A second group of 26 participants (mean age of 24 years old, 15 women and 11 men) was recruited for Phase 2 of the study. The sample size was chosen before data collection to have a minimum power of 0.8 with a one sample t-test, when a medium effect size (*Cohen’s d* = 0.5) is assumed. All participants were European-Canadian and had corrected-to-normal visual acuity.

#### 2.3.2 Procedure

The experiment consisted of six blocks of 100 trials (three blocks for Black faces and three blocks for White faces). Half of the participants were presented alternatively a block of White faces followed by a block of Black faces and vice-versa for the other half of participants. In each trial, two fully visible faces of the same race were presented side-by-side and the participants’ task was to decide which of the two was the most trustworthy. In half of the trials, the pair of faces selected comprised one face from the “high trustworthiness” category created in Phase 1, and another from the “low trustworthiness” category. In the other half of trials, the two faces were selected randomly from the three categories (i.e. “high”, “neutral”, and “low” trustworthiness) and were used as fillers for this experiment. This decision was made to increase the number of faces and thus, to avoid as much as possible that responses were based on memory rather than on the perception of trustworthiness for a given pair of faces. Only the trials comprising a “high trustworthiness” and a “low trustworthiness” face were included in the subsequent analysis. Note that randomly selected pairs that happened to include a low and high trustworthy target were not included in the analyses. Participants were told to respond quickly while avoiding errors. Each subsequent trial started approximately 300 ms after the participant’s response.

#### 2.3.3 Analysis and results

Except for two participants who had a performance of 55% and 56%, the large majority of participants performed well above the 50% chance level for discriminating between “high trustworthiness” and “low trustworthiness” faces, both for White (1st quartile = 79.9% and 3rd quartile = 88.1%) and Black faces (1st quartile = 73.2% and 3rd quartile = 83.0%). One sample t-tests confirmed that performance was above chance level for White (*M* = 81.66, *SD* = 9.73; *t*(25) = 42.8, *p*< .001, 95% CI [77.7, 85.6]) and Black faces (*M* = 76.87, *SD* = 9.99; *t*(25) = 39.2, *p*< .001, 95% CI [72.8, 80.9]). This suggests that the categories created in Phase 1 adequately represented trustworthy and untrustworthy faces for a large majority of individuals.

Participants scored higher when discriminating between the trustworthiness of White than Black faces (*t*(25) = 3.77, *p* < 0.001, 95% CI [0.02, 0.07], Co*hen’s d* = 1.55). However, we do not think that this difference was problematic for Phase 3. In fact, the main aim of this study was to verify if individual differences in racial prejudice are linked with the utilization of different facial information for trustworthiness judgments. If, for instance, the difference in performance is caused by trustworthy and untrustworthy White faces being easier to discriminate than trustworthy and untrustworthy Black faces, this difference can be considered as a constant across participants. Any modulation as a function of individual racial prejudice should therefore not be attributable to the difference in trustworthiness discriminability.

Results of Phase 2 confirmed that participants can discriminate with relatively high accuracy the trustworthiness of faces drawn from the “low trustworthiness” and “high trustworthiness” categories created in Phase 1. This verification was an important step, since Bubbles increase task difficulty by only making available a limited amount of visual information. To allow us to infer which facial information increases the probability of correctly discriminating faces’ trustworthiness, the Bubbles method used in Phase 3 relies on participants making errors related to information availability, over and above errors associated with the task difficulty when stimuli are completely revealed.

### 2.4 Phase 3

#### 2.4.1 Participants

A group of 75 participants (mean age of 23.5 years old, 57 women and 18 men) was recruited. The sample size was chosen before data collection to have a power of at least 0.8 with a Pearson correlation statistical test, assuming a medium effect size (*rho* = 0.3). All participants were White European-Canadian and had corrected-to-normal visual acuity. We had to remove one participant from the analyses because part of their data was lost.

#### 2.4.2 Stimuli

**Fig 2:**
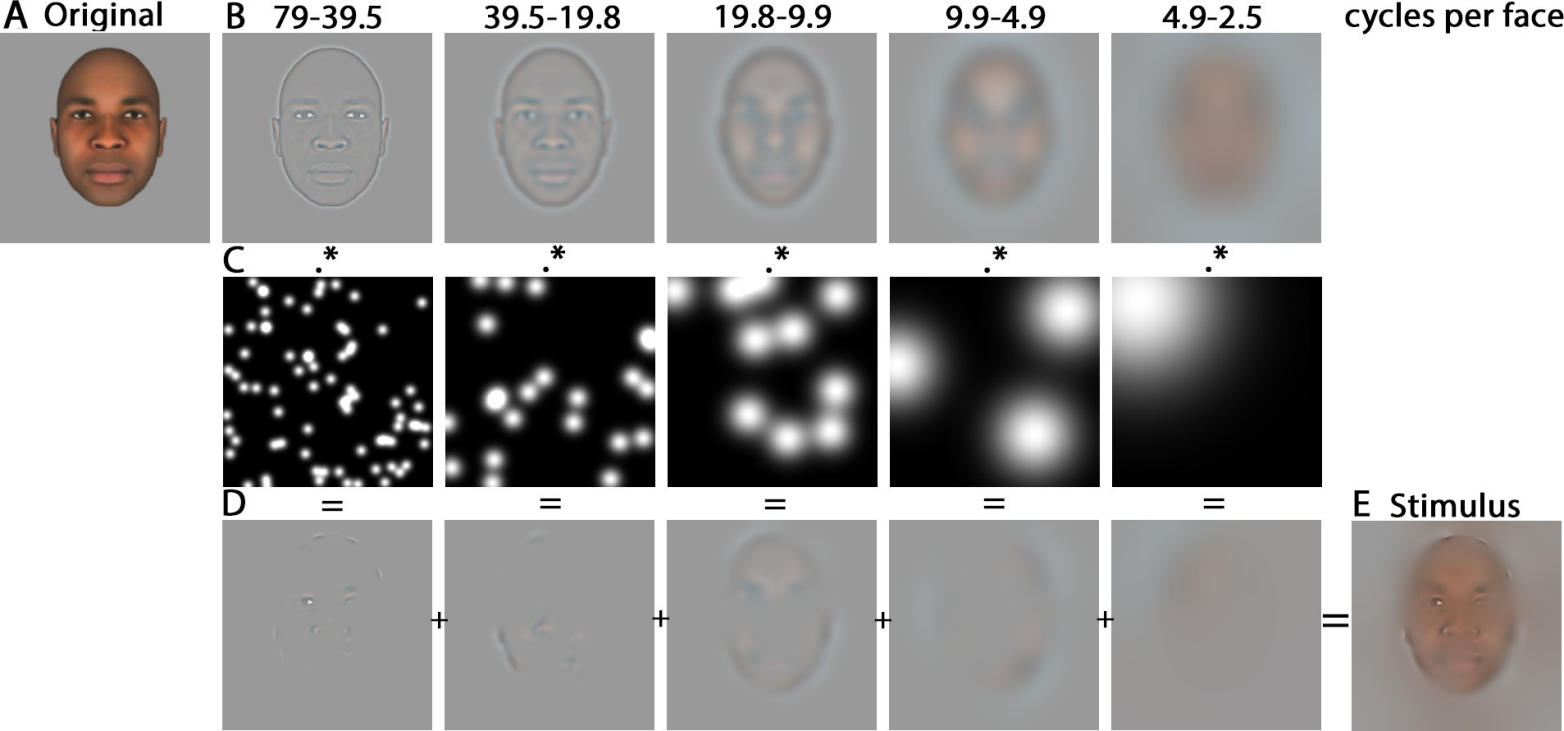
Illustration of the creation of a Bubblized stimulus. Each original stimulus (A) was first decomposed into five SF bands (B). Each filtered image was then independently sampled with randomly positioned Gaussian windows (i.e. Bubbles), so that sparse information is revealed (C). The information samples were summed across the five scales (D) to produce an experimental stimulus (E).

In this Phase, the stimuli were manipulated using the Bubbles method (for a demo of the method, see the link: https://osf.io/x6n25/). To create a Bubblized stimulus (see Fig 2), the picture of a face was first bandpass-filtered into five non-overlapping SF bands (SF; 128–64, 64–32, 32–16, 16–8, 8–4 cycles per image; or 79–39.5, 39.5–19.8, 19.8–9.9, 9.9–4.9, 4.9–2.5 cycles per face; the remaining bandwidth served as a constant background), using the Laplacian pyramid transform implemented in the pyramid toolbox for Matlab [42]. Afterward, each SF band was independently and randomly sampled using Gaussian apertures (or Bubbles) of varying standard deviations; that is, the size of the Bubbles was adjusted according to frequency band, consistently revealing 3 cycles of spatial information (standard deviations of the Bubbles were of 6, 12, 24, 48, and 96 pixels from the finest to the coarsest scale, respectively). Because the size of the Bubbles increased as the spatial scale became coarser, the number of Bubbles differed at each scale to keep constant the probability of revealing a given pixel in each SF band. Finally, the five randomly sampled images plus the background (the very low SF band; not represented in Fig 2) were summed to produce the experimental stimuli.

#### 2.4.3 Procedure

The experiment was divided into 10 blocks of 100 trials for each race of faces. Participants alternated between a block of trials with Black faces and a block of trials with White faces. The order in which the blocks were administered was counterbalanced across participants, such that half of them started with Black faces, and the other half started with White faces. On each trial, two Bubblized faces of the same race were presented side-by-side. The Bubbles’ location was identical on both faces to ensure that the available visual information was the same. As for Phase 2, in half of the trials, the pair of faces selected comprised one face from the “high trustworthiness” category, and another from the “low trustworthiness” category. The other half of trials represented filler items, and the two faces were selected randomly from the three categories (i.e. “high”, “neutral”, and “low” trustworthiness). Only trials that included both a “high trustworthiness” and a “low trustworthiness” face were included in the subsequent analysis. Note that randomly selected pairs that happened to include a low and high trustworthy target were not included in the analyses. Participants were asked to decide which of the two faces appeared the most trustworthy. They were told to respond as quickly and accurately as possible. The next trial started approximately 300 ms after the participant’s response. The average accuracy of each participant with each race of faces was maintained at 65% (halfway between chance and performance with whole faces) by adjusting the number of Bubbles on a trial-by-trial basis using QUEST [43]; more Bubbles implies that more information is available to carry out the task. Thus, the number of Bubbles can be conceived as a measure reflecting the relative ability of the participants (i.e. the better someone is at processing faces, the fewer number of Bubbles/facial parts they need to accurately carry out this task [44]). Finally, after completing all blocks with each race, a race IAT [36] was administered to all participants to measure their level of implicit racial bias towards Black versus White individuals. The IAT was administered following the procedures of Greenwald, Nosek, & Banaji [45]. In order to measure the strength of automatic associations, participants are asked to categorize as fast as possible White and Black faces as well as pleasant (e.g. love, peace, joy) and unpleasant words (e.g. war, horror, terrible) using a keyboard. In one condition, White faces and pleasant words were associated with the same key whereas Black faces and unpleasant words were associated with another key. In a second condition, White faces and unpleasant words were associated with the same key whereas Black faces and pleasant words were associated with another key. Implicit biases were then calculated based on reaction times for correct items by computing the difference between the two conditions.

#### 2.4.4 Analysis and results

2.4.4.1 Average information used for trustworthiness judgment with White and Black faces. A paired t-test was first conducted on the number of Bubbles necessary to maintain an average performance of 65% with White (*M* = 87.83 Bubbles, *SD* = 83.68) and Black faces (*M* = 123.71 Bubbles, *SD* = 83.19). Participants needed significantly less visual information to perform the task with White than with Black faces (*t*(73) = -3.78, *p* < .001, *Cohen’s d* = .43, 95% CI [-54.78, -16.98]). A paired t-test was also conducted on reaction times with White (*M* = 1.31 second, *SD* = .76) and Black faces (*M* = 1.28 second, *SD* = .50). No significant difference was found (*t*(73) = 0.27, *p* = .79, *Cohen’s d* = 0.03, 95% CI [-.14, .18]).

To uncover the facial information used by observers to accurately discriminate faces based on their trustworthiness, we conducted what amounts to a least-square multiple linear regression on the location of the Bubbles (i.e. pixel locations on which each Bubble was centered in each SF band) and the accuracy of the judgment made. More precisely, a weighted sum of the Bubbles mask was calculated by allocating Bubbles positive or negative weights when they led to correct or incorrect responses, respectively. The values of the positive and negative weights were obtained by transforming the accuracy (1 for a correct answer; 0 for an incorrect answer) into z-scores (using the average accuracy and standard deviation calculated across all trials). This operation yielded what is called a classification image (CI): it reveals which facial regions in each SF band are systematically associated with an accurate trustworthiness decision. The individual CI in each SF band were smoothed using a Gaussian filter with a standard deviation corresponding to the ones used during the experiment. The individual CI were then transformed into z-score values, using a permutation procedure to estimate the mean and the standard deviation under the null hypothesis. The resulting CI were finally summed across all participants to create a group CI, and divided by the square-root of the number of participants. To determine what visual information significantly correlated with accuracy, we used the Cluster test from the Stat4CI toolbox (for details see [46]). The statistical threshold provided by this test corrects for multiple comparisons.

**Fig 3:**
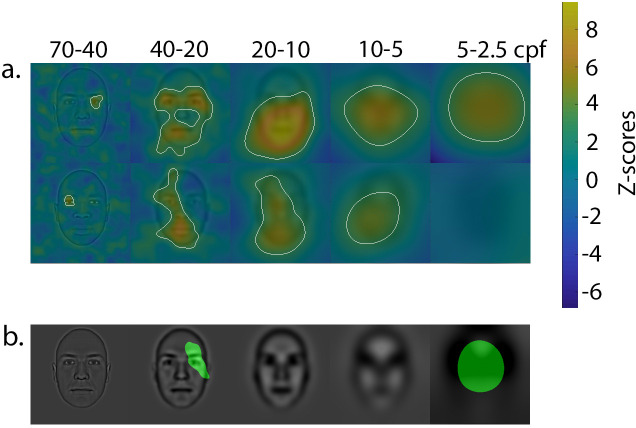
a) Facial areas correlated with accuracy for trustworthiness judgments of White (top row) and Black (bottom row) faces in each SF band. The colors represent the z-score value reached on each pixel; the higher the z-score value, the higher the association between the use of that pixel and accurate trustworthiness judgments. The areas circled in white are those that reach the significance threshold for a given racial group. b) The areas highlighted in green were significantly more used with White than with Black faces. No area was more used with Black than with White faces.

The results are displayed in Fig 3. Note that in all of the following descriptions, references to the left or right sides of the face are from an observer’s point of view. For White faces, accurate trustworthiness judgments were correlated with the use of the right eye/eyebrow area in the highest SF band, the use of both eyes/eyebrows and the mouth area in the second SF band, and with the utilization of all internal features in the three lowest SF bands. For Black faces, accurate trustworthiness judgments were correlated with the use of the left eye/eyebrow area in the highest SF band, the use of the left eye/eyebrow and mouth areas in the second SF band, and the utilization of all internal features in the third and fourth SF bands; no facial features reached statistical significance in the lowest SF band. Two facial areas were statistically more correlated with accuracy for White than Black faces: the right eye/forehead area in the second SF band, and all internal features in the lowest SF band. It should be noted that although some facial regions are significantly correlated with accurate judgments of trustworthiness for one specific racial group of faces (and not the other), one cannot infer that these regions are systematically more used for this group of faces than the other. Indeed, some regions may reach the significance threshold for a particular region for one race, but be just below the threshold of significance for the other, resulting in an absence of significant difference between the two races of face.

2.4.4.2 Effect of implicit bias on facial information used for trustworthiness judgments. Participants’ implicit bias was calculated by computing D scores, using the procedure described by Lane, Banaji, Nosek, & Greenwald [47]. Negative D score values indicate a pro-White/anti-Black bias which means that participants were faster to respond to White faces when associated with positive words and to Black faces when associated with negative words. On average, participants obtained D scores of -0.658 (*SD* = 0.45). The D scores’ first and third quartiles were of -0.97 and -0.35, respectively. Thus, the majority of participants had a pro-White/anti-Black bias.

To begin with, the correlation between the number of Bubbles necessary to correctly perform the task (which represents an index of ability with respect to the task) and implicit bias was calculated. No such correlation reached significance (White faces: *r* = -.072, *p* = .54; Black faces: *r* = -.05, *p* = .69). Moreover, no correlation was found between the difference in the number of Bubbles required with Black and White faces and implicit bias (*r* = -.026, *p* = .83), and none was found between reaction times for trustworthiness judgments and implicit bias (White faces: *r* = .04, *p* = .71; Black faces: *r* = .05, *p* = .65), nor between the difference in reaction times for Black and White faces and implicit bias (*r* = .008, *p* = .94).

**Fig 4:**
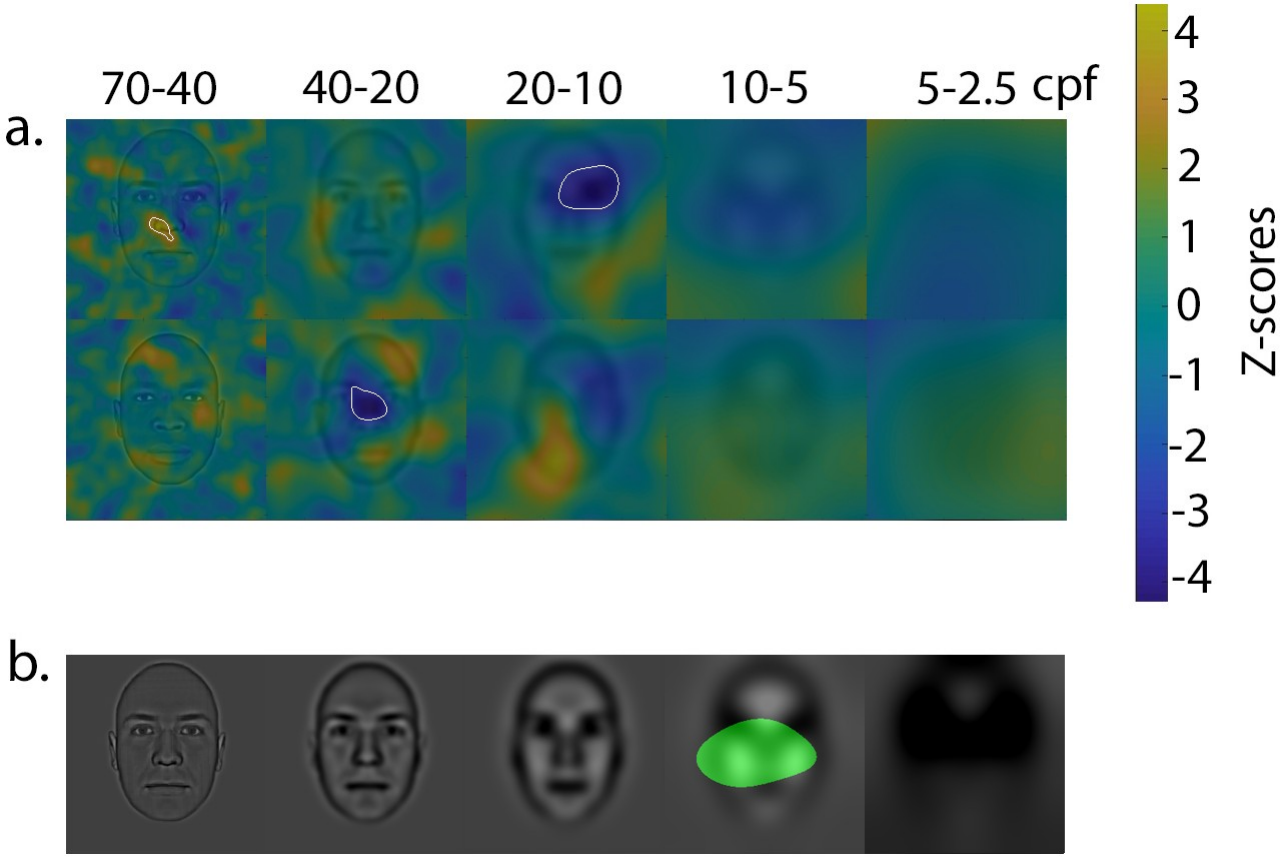
a) Association between implicit racial bias and the facial information used for trustworthiness judgments. The different colors represent the degree of association (in z-scores) between implicit racial bias and the utilization of facial information. Yellow indicates a positive association between D scores and information use; in other words, areas represented in yellow were more used by individuals with lower pro-White/anti-Black biases. Dark blue indicates a negative association between D scores and information use; in other words, areas represented in dark blue were more used by individuals with higher pro-White/anti-Black biases. The areas circled in white are the ones for which the association reached the significance threshold. b) The area highlighted in green is the one for which a differential use of information with White and Black faces was associated with implicit racial bias. More specifically, individuals with a larger pro-White/anti-Black bias made more use of the area depicted in green with White than with Black faces.

In order to assess whether the diagnostic information for trustworthiness judgments changes as a function of individual variations in implicit bias, a CI representing the association between the facial information used by each participant and their D score was computed. After transforming D scores into z-score values, we computed a weighted sum of the individual’s CI using these z-scores as weights. These weighted CI were then transformed into z-score values using the area corresponding to the background of the stimuli (i.e. the area containing no face signal whatsoever) as a measure of the mean and standard deviation of the null hypothesis. Again, we used the Cluster test from the Stat4CI toolbox to find a statistical threshold that took into account the multiple comparisons. This procedure was followed separately for each race. The results are displayed in Fig 4. For White faces, the higher the level of implicit bias, the less likely participants were to use the nose in the highest SF band, and the more likely they were to use the upper part of the face in the third SF band. For Black faces, the higher the level of implicit bias, the more likely participants were to use the upper part of the face in the second highest SF band. Most importantly, implicit biases were associated with different uses of lower SF (i.e. the fourth SF band) as a function of race: namely, the higher the level of implicit bias, the more likely participants were to use the upper part of the face with White faces, but the less likely they were to use this information with Black faces. Although this difference only reached significance in the fourth SF band, the same trend can be observed in the lowest SF band (i.e. the fifth SF band), where the maximum difference in z-score between Black and White faces reaches 2.48, a value that would reach the statistical threshold with the Pixel test from the Stat4CI toolbox. The Pixel test also compensates for the multiple comparisons, but for the sake of homogeneity, we used the same test (i.e. the Cluster test) throughout all of the analyses. The same results were obtained when transforming the D scores into ranks rather than z-scores (akin to a Spearman correlation).

## 3. Discussion

More and more studies are taking an interest in the interaction between social cognition and visual perception (e.g. [48,49]). Until recently, visual perception was conceived–implicitly or explicitly–by most researchers as universal (see [50] for a similar argument) and encapsulated [51] from other higher-level processes. Since then, evidence has accumulated showing that visual perception in general, and visual extraction strategies in particular, are modulated both by external influences such as culture [50,52–55], and by personal characteristics such as personality traits (e.g. [56]) and perceptual skills (e.g. [44,57–61]). The main aim of the present study was to investigate the impact of implicit racial attitudes on the visual extraction strategies used during a face trustworthiness comparison task.

### 3.1. Difference in performance and visual information when discriminating between the trustworthiness of White and Black faces

As mentioned in the results section of Phase 2, participants scored higher when discriminating between the trustworthiness of White than of Black faces. This difference in terms of task difficulty translated into participants needing, in Phase 3, significantly less visual information to perform the task with White than with Black faces. This finding goes along with other studies demonstrating that White observers have more difficulty discerning if a Black individual compared to a White one is lying or being truthful [62,63]. Struggling to discern truths from lies in an interracial context could lead to unwillingness towards Black people and ultimately increase racism. Experiencing more difficulty to judge trustworthiness of Black individuals can also have serious practical implications in situations where one has to decide quickly to trust someone or not. For example, racial shooter biases have been documented in laboratory settings with shooting tasks, and worse, in a real-life context such as extrajudicial police shootings of minority ethnic group members (see [64] for a meta-analysis).

### 3.2 Association between implicit racial bias and the visual information underlying trustworthiness judgments

The results indicated an association between implicit racial bias and the visual information underlying trustworthiness judgments. More specifically and depicted in Fig 4B, a large part of the face (including the top of the lips through the middle of the forehead) in the second lowest SF band was differentially used in White and Black faces as a function of implicit bias. A trend in the exact same direction was also observed in the lowest SF band. More precisely, individuals higher in pro-White/anti-Black bias relied significantly more on low SF with White than with Black faces. To the best of our knowledge, the present study is the first to reveal a direct link between implicit racial bias and low-level visual information (such as SF) extraction.

The finding that lower SF were more useful with White than Black faces, and that this differential use of information was modulated by implicit racial bias, may be congruent with studies that have revealed that configural processing influences the perceived humanness of a face [65,66]. On the one part, many studies have shown that White individuals have a tendency to dehumanize Black individuals (see [67] for a review). On the other part, it has been proposed that configural processing is mostly supported by the processing of low SF [68–70]; see however [71]. Configural processing has also been proposed as crucial during trustworthiness judgments of faces [7]. Interestingly, a recent study showed that it was harder for participants to punish own-race faces displayed in low spatial frequency [72]. Taken together, these results could suggest that lower reliance on low SF with Black faces, as observed in the present study, may reflect a lower reliance on configural processing during trustworthiness judgments of Black faces, especially in individuals with higher implicit racial biases. It is noteworthy to mention that Black people are not the only dehumanized group. For example, Fincher and Tetlock [72] suggest that people process the faces of norm violators, another kind of dehumanized group, differently–i.e. without the use of face-specific processes. Evaluating if the modulation we observed in the present study generalizes to a different social (but same-race) group could be the focus of an interesting future study.

Another potential explanation for the higher use of low SF with White than with Black faces is that the facial features mostly coded in low SF affect the babyfaceness appearance. Babyfaceness has been shown to be strongly associated with trustworthiness perception [73,74]. The skin of faces displayed in low SF appears very smooth; the wrinkles and skin spots often associated with aging are best coded by higher SF (see Figs 6 and 7 from [13]). Moreover, baby faces are characterized by round shape faces, and the global face shape is best coded by low SF. Besides, studies have shown that Black boys are perceived as older and less innocent than their White peers [75]. Thus, it is possible that when judging face trustworthiness, White individuals (especially those with higher implicit racial biases) rely less on features reflecting babyfaceness in Black faces, those features being mostly coded in low SF.

Note that other facial areas were also correlated with implicit bias, but their utilization was not differentially modulated as a function of race. As explained in the results section, the utilization of a facial area may come out as significantly associated with one race but not with the other, and one must be careful with the interpretation of such findings. For instance, the upper part of Black faces came out as significantly associated with implicit biases in the second SF band, indicating that the higher the pro-White/anti-Black bias, the more this area is used with Black faces. However, it did not come out as significantly more associated with Black than with White faces, indicating that the association was likely in the same direction but weaker with White faces. The same holds true for the finding that with White faces, the nose in the first SF band and the upper part of the face in the third SF band were associated with implicit biases. These areas did not come out as significantly more associated with White than Black faces. Nonetheless, it is interesting to note that in Black faces, the areas most linked to implicit racism are located at the level of the frown in the mid-high spatial frequencies. The frown is particularly diagnostic of the expression of anger [10], an expression inversely associated with the perceived level of trustworthiness [8]. Instead, with White faces, implicit racism is mostly associated with the utilization of the eyes/eyebrows area, which is a diagnostic area in face identification [60] and probably very important for triggering a humanizing mode of face perception (see [76]). The observation that the most prejudiced individuals rely more on features typically containing anger information is particularly surprising given that connectionist modeling has revealed that Black faces contain fewer angry features than White faces [77]. Once again, this supports the idea that implicit biases modulate the use of information in faces in a way that may induce the perception of anger/untrustworthiness, even when anger is not the most salient information.

### 3.3 Limits of the present study

As explained above, the main aim of the present study was to investigate the potential impact of implicit racial bias on the visual strategies underlying trustworthiness judgments. However, another question of interest in the field is the degree to which individuals rely on the same strategies to judge trustworthiness with faces of their own- vs. other-race group, with no regard to implicit bias. Because the present study included a homogeneous sample of participants with regard to race (i.e. White participants), more studies will be necessary to fully understand if (and how) the visual strategies underlying trustworthiness judgments vary for own vs. other race faces. In fact, the present results could reflect different mechanisms that cannot be distinguished from one another based on a sample solely composed of White individuals. For instance, it may reflect a general mechanism in which the processing of own-race faces relies more on lower SF. Alternatively, it may reflect a more specific mechanism whereby the different physiognomies of White and Black faces induce visual strategies that are best adapted to the facial features of each race. Another possibility is that the differences observed in visual strategies for Black vs. White faces are not related to race per se, but to other factors such as high vs. low status groups or majority vs. minority groups. In fact, Black individuals have been associated with low-status whereas White individuals have been associated with high-status [78]. Collecting data with a sample of Black participants would help disentangle the three possibilities.

One potential limit of this study is the criterion we used to divide faces in three trustworthiness categories. More specifically, we divided faces based on their rating ranks (i.e. the 50 faces with the highest trustworthiness level, the 50 faces with the lowest trustworthiness level, and the other faces considered neutral or difficult to discriminate on this trait) rather than on their absolute ratings. This led to categories that were slightly different, in terms of average ratings, for Black and White faces. However, we think that the categories created using ranks more closely reflect ecological variations in trustworthiness evaluations. Moreover, we do not think that this difference alters the interpretation of the link between information utilization and racial biases, since the categories represented a constant across participants. However, it may impact our interpretation of the average strategy (i.e. feature in SF utilization regardless of biases) used with Black vs. White faces, i.e. the results presented on Fig 3.

Another potential limit is that racial biases were not controlled in Phases 1 and 2. However, the aim of these two Phases was solely to create the highly trustworthy and highly untrustworthy face categories, and we do not think that racial biases have interfered with the process. The fact that the inter-individual correlations were as high for Black than for White faces suggests that the ranking of faces was not affected by racial biases. In fact, we would have expected lower inter-individual correlations for Black than for White faces if racial biases had affected the ranking. Furthermore, we report inter-individual correlations that are very similar to previous studies involving only White faces (e.g. [41]), again suggesting that the ranking of faces was not affected by racial biases. For Phase 2, we believe that categories were adequate since performance was above chance level. Finally, in Phase 3, to verify if our face categories were adequate, we analyzed the number of Bubbles required to succeed at the task, since the number of Bubbles is a measure that reflects the ability to perform a task (see [44]). The more difficult a task, the greater the number of Bubbles required. If face categories had been affected by implicit bias, we would have expected some variations in performance between individuals with high and low implicit bias with respect to the number of bubbles in Phase 3. However, we did not find any correlations between the number of bubbles and the level of racial prejudices.

Nonetheless, future studies are needed to replicate these findings with new face stimuli, or perhaps with computer-generated faces. However, even if computer-generated faces seem like an interesting approach to minimize differences in trustworthiness across racial stimuli, some important concerns must be taken into account regarding their use. For instance, a recent study provides some evidence that computer-generated faces are not processed in the same way as real faces for trustworthiness judgments [79]. Moreover, computer-generated faces usually do not control for the fact that different facial characteristics across races might be related to the perception of trustworthiness.

Finally, all face stimuli used in the present study were from male individuals. As just explained, facial physiognomy may affect the visual strategies deployed to extract the information necessary for trustworthiness judgments; the different physiognomy of male and female faces could thus lead to the utilization of slightly different strategies. Moreover, stereotypes and attitudes may also affect the way in which male and female faces are processed. For instance, it has been shown that male and female faces that are similar in terms of a facial trait dimensions (e.g. similar in terms of trustworthiness) are evaluated differently in terms of valence [80]. Additionally, implicit bias about gender and race may interact; needless to say, more research needs to be done to better understand the impact of gender, race, and implicit attitudes on the extraction of visual information during trustworthiness judgments.

## Conclusion

To summarize, the present study examined whether implicit racial biases modulate the visual information used to judge trustworthiness of own- vs. other-race faces. Interestingly, low SF, which refer to coarser visual information, were more used with White than with Black faces to make an accurate trustworthiness judgment. Moreover, this differential use of low SF with White and Black faces was correlated with implicit racial biases. This pattern of results may reflect a higher reliance on configural processing or a stronger tendency to search for babyfaceness cues with White than with Black faces, especially in individuals with higher implicit racial biases.
